# Regional Rainfall Warning System for Landslides with Creep Deformation in Three Gorges using a Statistical Black Box Model

**DOI:** 10.1038/s41598-019-45403-9

**Published:** 2019-06-20

**Authors:** Zizheng Guo, Kunlong Yin, Lei Gui, Qingli Liu, Faming Huang, Tengfei Wang

**Affiliations:** 1 Faculty of Engineering, China University of Geosciences, Wuhan, 430074 China; 2 Chongqing Wanzhou Institute of Geological Environment Monitoring, Chongqing, 404000 China; 3 School of Civil Engineering and Architecture, Nanchang University, Nanchang, 330000 China; 4 Institute of Geological Survey, China University of Geosciences, Wuhan, 430074 China

**Keywords:** Natural hazards, Hydrology

## Abstract

Establishing an efficient regional landslide rainfall warning system plays an important role in landslide prevention. To forecast the performance of landslides with creep deformation at a regional scale, a black box model based on statistical analysis was proposed and was applied to Yunyang County in the Three Gorges Reservoir area (TGRA), China. The data samples were selected according to the characteristics of the landslide displacement monitoring data. Then, the rainfall criteria applied to different time periods were determined by correlation analysis between rainfall events and landslides and by numerical simulation on landslide movement under certain rainfall conditions. The cumulative rainfall thresholds that were determined relied on the displacement ratio model, which considered landslide scale characteristics and the statistical relationship between daily rainfall data and monthly displacement data. These thresholds were then applied to a warning system to determine a five-level warning partition of landslides with creep deformation in Yunyang County. Finally, landslide cases and displacement monitoring data were used to validate the accuracy of the model. The validation procedure showed that the warning results of the model fit well with actual conditions and that this model could provide the basis for early warning of landslides with creep deformation.

## Introduction

Landslides are among the most serious geological disasters in China, as well as other Asian countries^1–3^. There have been over 3800 landslides in the Three Gorges Reservoir area (TGRA), China, since the TGRA was first impounded in 2003^4,5^, and due to the wide area of distribution, high frequency, and fast movement^6,7^, these landslides create significant threats to local residents every year^8,9^. Therefore, a comprehensive risk management strategy is needed to reduce disaster risks^10,11^. As part of a sustainable management plan for disaster risks, the installation of an early warning system is often a cost-effective risk mitigation measure^12^.

Rainfall is one of the major external forces triggering landslides in the TGRA^13–15^. Thus, how to provide early warning for landslide hazards under heavy rainfall conditions has always been a popular issue^16,17^. Numerous models have been developed to provide landslide warnings based on rainfall amounts, and can generally be divided into two categories: physical approaches based on model testing or numerical simulation^18,19^, and statistical black box models^20^. Since physically based approaches can only be effectively applied over small sites^21^, at a regional scale, the most common methodologies used are black box models based on empirical or statistical rainfall thresholds^22^. The majority of black box models are based on empirical or statistical studies of the rainfall characteristics that have led to the onset of landslides in the past^23,24^. Such studies are aimed at using rainfall monitoring data to establish a statistical relationship between the temporal and spatial distribution of landslides and rainfall characteristics^25,26^. Therefore, other complex physical processes involved in landslides are often ignored, since it is too difficult to correctly account for these processes over large areas^22^.

Choosing the appropriate criteria for defining rainfall thresholds is necessary to establish an accurate rainfall warning system for landslides. The intensity (*I*) and duration (*D*) of rainfall are the most commonly utilized criteria^25,27,28^. For example, Caine^29^ was the first to establish an empirical power law between *I* and *D* to define a lower boundary of the rainfall conditions associated with shallow landslides. In addition, cumulative rainfall is also widely used^26,30^, as well as antecedent rainfall conditions and other thresholds, including hydrological thresholds^23,31^. However, current research generally considers the conditions that have initiated landslides in the past as dependent variables in statistical black box models^28,32^, and these results are more applicable to presliding warnings. Apart from landslides which were induced suddenly, there are many landslides with creep deformation that exist in the TGRA, with annual displacements from tens to hundreds of millimeters, but few researchers have focused on the rainfall thresholds for these. For this type of landslide, short-term acceleration of deformation after heavy rainfall still poses a serious threat to the lives and properties of residents on the landslide^33^. Thus, even when slow landslides are associated with a low destructive significance, monitoring and early warning remain essential for hazard management^34^.

The aim of this paper is to establish a rainfall warning system applicable to landslides with creep deformation at a regional scale, with Yunyang County in the TGRA being selected as the study area. The excellent professional monitoring system in place provides us with adequate monitoring data as the base of our statistical methods. A statistical black box model was first established to obtain critical rainfall thresholds and then these thresholds were applied within a warning system. Finally, the real-time rainfall data and landslide deformation monitoring data were selected for system validation.

## Study Area

### Geological conditions

**Figure 1:**
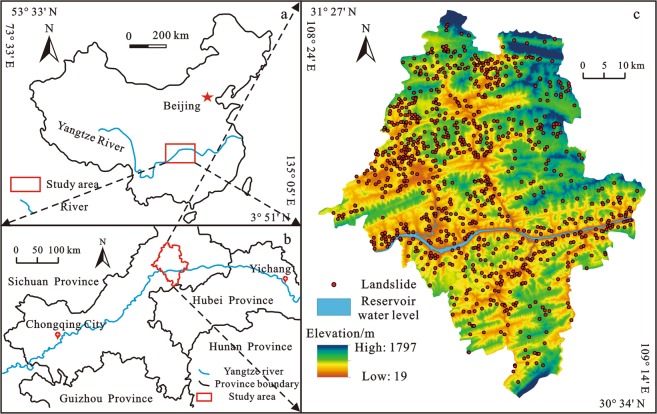
The location of Yunyang County and distribution of the landslides with creep deformation: (**a**) Location of the TGRA; (**b**) Location of Yunyang County; (**c**) Distribution of landslides with creep deformations in Yunyang County. (The maps were created by Zizheng Guo using ArcGIS 9.3, http://www.esri.com/).

Adequate monitoring data is the basis of the statistical research. Previous studies show that the amounts of landslides in Wanzhou District, Yunyang County, Fengjie County and Wushan County are the largest in the TGRA^35,36^. Among them, the monitoring system of landslides in Yunyang County are more developed and the amount and volume of landslides are larger, so Yunyang County was taken as the study area. It is located in northeast of Chongqing City, on the upper Yangtze River, with a total area of 3649 km^2^. It is bordered by Wanzhou District, Kai County, Fengjie County, Wuxi County and Lichuan City, Hubei Province^6,37^ (Fig. 1). The region is located at the eastern edge of the Sichuan Basin, and it is a transitional zone between hills and mountains. The geological structures are dominated by ejective folds, and the fault scales are small. The terrain is similar to the rhombus with high elevation in the north and south, and low elevation in the middle. The strata mainly include the Quaternary, Jurassic and Triassic, among which the sandstone and mudstone of the Jurassic are the most widely distributed^38,39^.

Yunyang County has a typical subtropical monsoon climate, with mild, dry winters and hot summers with increased rain. The average annual rainfall is 961.2 mm, and rainfall is mainly concentrated between May and September, with significant spatial differences^38,39^. The surface water system in the territory is developed, and the Yangtze River, along with dozens of tributaries and small-scale creeks, constitute a complex surface runoff network^35^.

**Figure 2:**
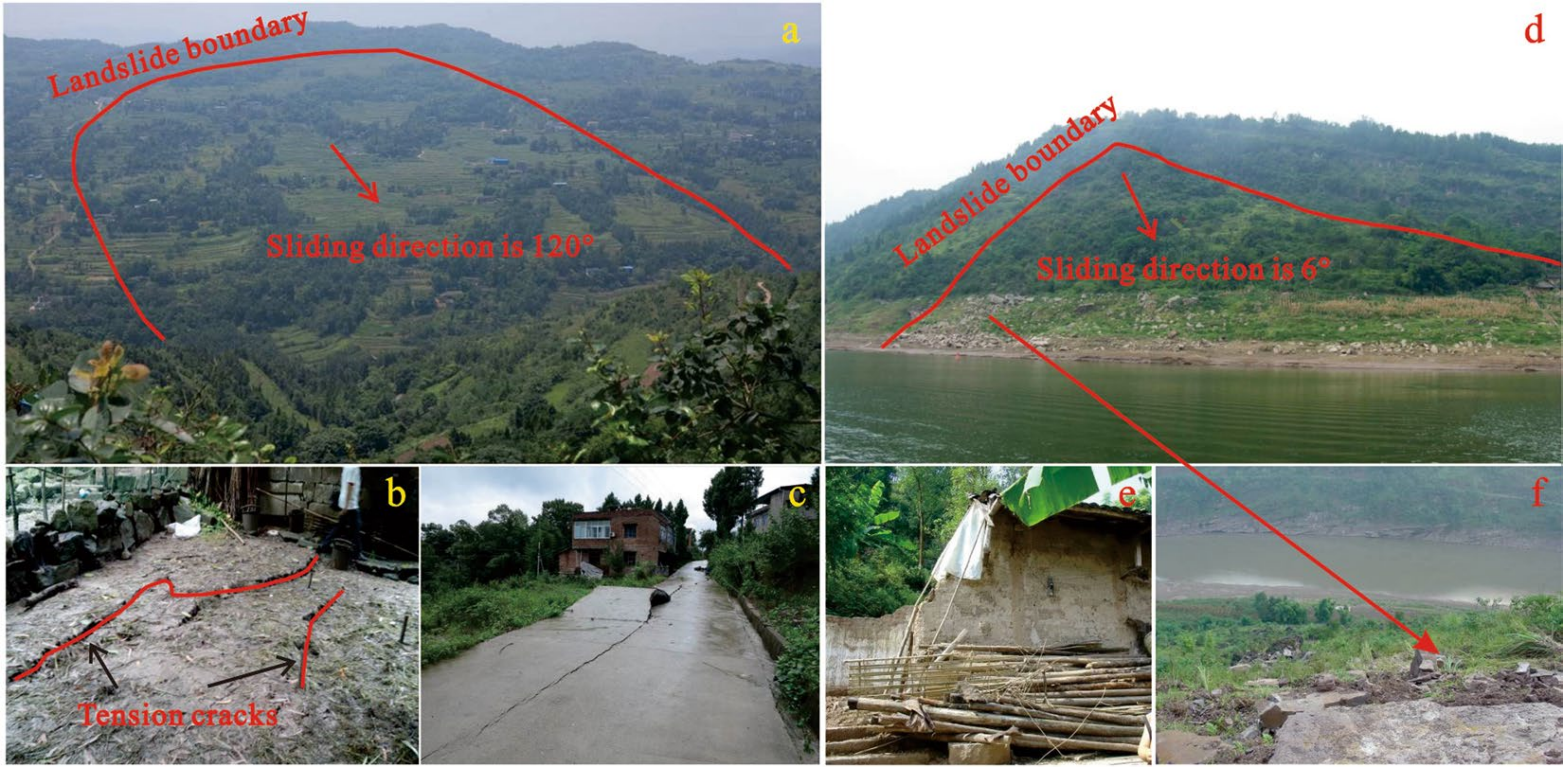
The photos of different types of landslides in the study area: (**a**) The topography of CJBL. (**b**) The tension cracks on the ground of CJBL. (**c**) The uplift of road on CJBL. (**d**) The topography of BJSL. (**e**) The damaged house caused by BJSL. (**f**) The small-scale sliding on the toe of BJSL.

There are 971 landslide hazards that have been identified in the study area, accounting for 95% of the total geological hazards in the region, and the amount of landslides with creep deformation is 691. Influenced by the combined internal conditions and triggering factors, their deformation are continuous but very slow under constant stress. The total area of these landslides is 71.3 km^2^ and the cumulative volume is 0.68 billion m^3^, including 11 landslides with volume more than 10 × 10^6^ m^3^ and 232 landslides with volume ranging from 10 × 10^5^ m^3^ to 10 × 10^6^ m^3^. Due to the characteristics of large quantity and large volume, these landslides have caused serious disasters to the lives and properties of residents. Since the impoundment of the TGRA, hundreds of thousands of people have been threaten, including hundreds of deaths, many missing persons and billions of economic losses. Among these landslides, most landslides consists of engineering soil, such as silty clay with a small amount of rubbles. Small amount of landslides consist of bedrock (sandstone and mudstone of Jurassic). So, from the perspective of the types of materials, these landslides can be divided into earth slide and rock slide. The examples of different types were showed in Fig. 2. The Caijiaba landslide (CJBL) is a typical earth slide with an area of 18 × 10^5^ m^2^ and a volume of 2 × 10^7^ m^3^. The material of it is silty clay with a small amount of rubbles, overlaying the bedrock composed of sandstone and mudstone. The CJBL was activated by the rainstorm on September 2000 and many tension cracks and road uplift were observed on the crown of the landslide, causing threats to hundreds of residents on the landslide. The materials of Bijianshan landslide (BJSL) compose of sandstone and mudstone of Jurassic, which has an area of 15 × 10^4^ m^2^ and a volume of 3 × 10^6^ m^3^. Due to the bedrock is the main component of the materials, BJSL is considered as a rock slide. It was induced by rainfall on September 1998 and some houses were damaged. In recent years, under the influence of reservoir water level, the stability of BJSL is worse and the small-scale sliding occurred on the toe of it.

### Landslide professional monitoring system

**Figure 3:**
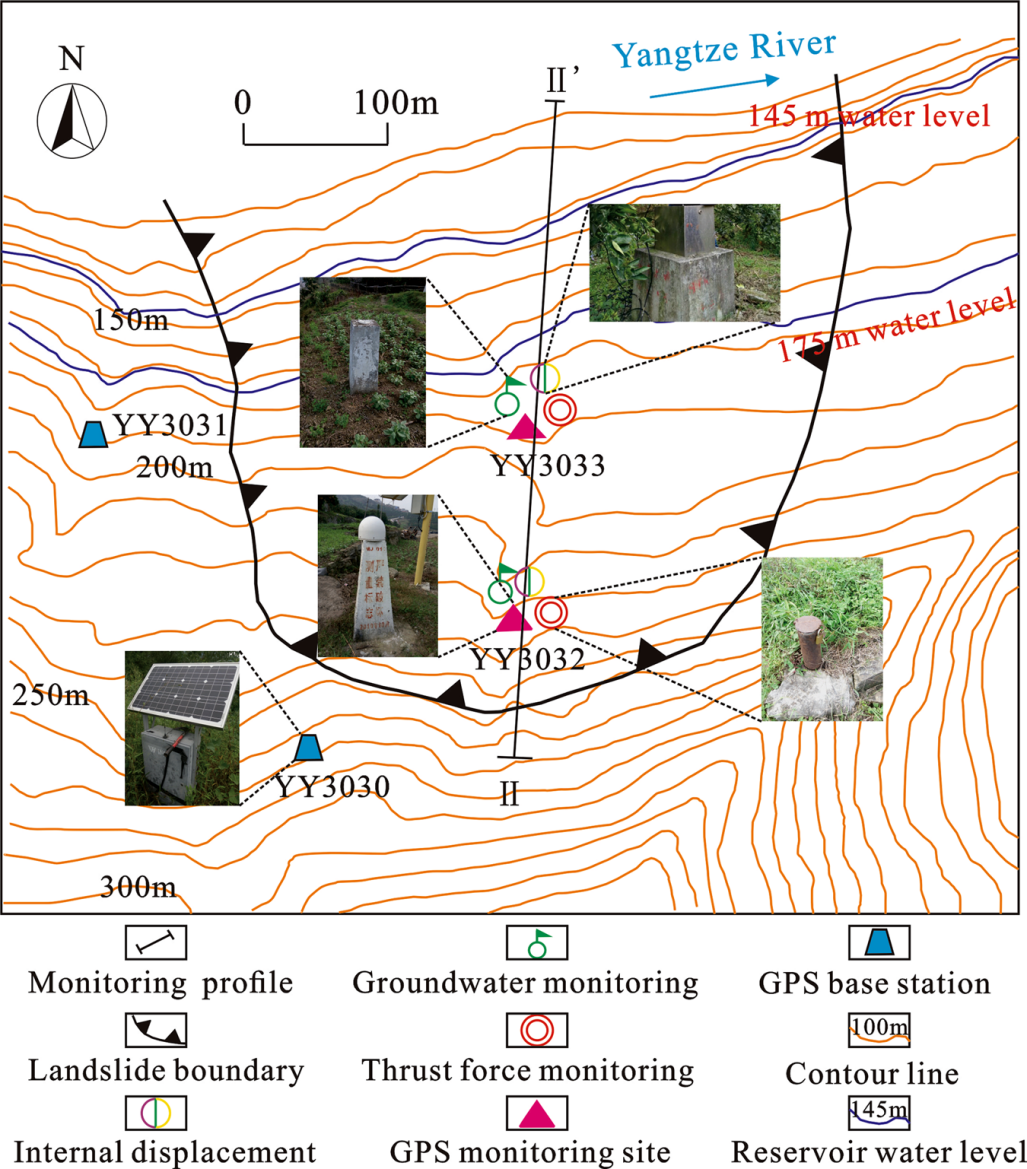
A diagram of the monitoring system in a landslide in Yunyang County (The figure was created by Tengfei Wang using CAD 2014 (https://www.autodesk.com.cn/) and CorelDRAW X7 (https://www.corel.com/cn/)).

Traditionally, there are two common methods for landslide monitoring and early warning: professional monitoring systems^5,37^ and community-based disaster risk reduction (CBDRR) systems^5^. Professional monitoring systems use a variety of integrated monitoring methods to implement omnidirectional landslide monitoring at the surface and at depth, such as groundwater monitoring, GPS surface displacement, internal displacement, and rainfall and thrust force monitoring (Fig. 3). Professional monitoring systems are characterized by high precision, a high automation degree, and a wide array of varieties. They can provide a range of monitoring data to meet the needs of risk management. Currently, there are a total of 254 geological disaster professional monitoring sites in the TGRA. Among these sites, there are 27 landslides in Yunyang County.

## Rainfall Thresholds

### Data sources

Different types of data are applicable for different types of landslides, so monitoring data of different types of landslides must be distinguished. The creep is simply deformation that continues under constant stress and it exhibits the typical “three-stage” characteristics only under the weight of landslide^40^. However, due to the disturbance of external influencing factors, the deformation curve will eventually deviate from the ideal curve. If the landslide is affected by nonperiodic factors (e.g., human activities, earthquake, etc.), the displacement curve displays an undulating oscillation. Under the influence of periodic factors (e.g., normal reservoir water level schedule, etc.), the displacement curve tends take on a “step-like” form^15,33,40^. In addition, the cumulative displacement may remain at a similar level due to the stable state of landslides or errors in the monitoring process.

**Figure 4:**
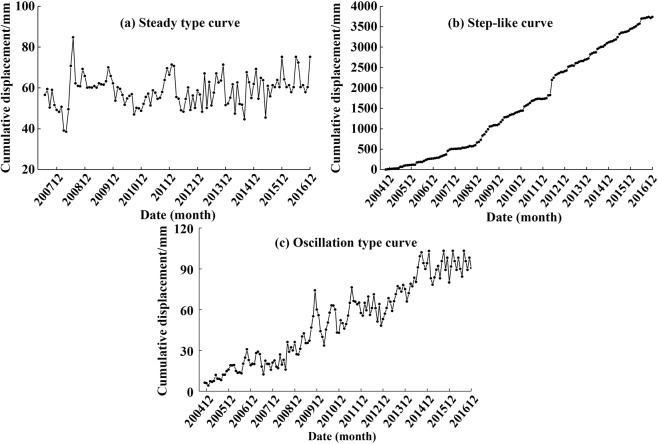
Different kinds of cumulative displacement curves of landslides: (**a**) YY3039 monitoring site on Dashiban landslide (DSBL). (**b**) YY025 monitoring site on Huangniba landslide (HNBL). (**c**) YY127 monitoring site on Meiziba landslide (MZBL).

The monitoring data was gathered from the archives of the Institute of Geological Environment Monitoring in Yunyang County. These long-term data indicated that the deformation of each landslide was continuous, and the average velocities of all monitoring points shows all landslides performed monitoring system belonged to the class of “extremely slow” or “very slow”. So the deformations of landslides are creep^41,42^. In addition, it should be noted that for very slow and extremely slow landslides, monthly displacement data can be considered detailed enough. As seen in Fig. 4, all of the above three displacement curves can be found in this area. Sometimes due to the result of accidental errors or spatial inhomogeneity of landslide deformation, different types of displacement curves may appear at different monitoring points on the same landslide. However, a study regarding landslide rainfall warnings should primarily focus on landslide movements during or after rainfall events. It is not practical to incorporate landslide displacement data into statistical samples without significant accelerated deformation. Therefore, to eliminate the disturbance of landslides with stable deformation, statistical samples with similar deformation characteristics are first selected from each landslide. The principles for selection require obvious deformation trend of monitoring data in recent years. Meanwhile, considering the systematic errors associated with GPS equipment, the monitoring points with an average annual displacement component of less than 10 mm along the main sliding direction during the entire monitoring period are eliminated. In total, displacement data from 79 monitoring systems was kept to construct the final analysis sample, which accounts for 45% of all monitoring systems.

### Division of study period

Rainfall and reservoir water level fluctuation are the two most important factors triggering landslide movement in the TGRA^40,43^. We can only study the impact of rainfall on landslides separately by properly controlling for the reservoir water level fluctuation. Therefore, it is necessary to determine a reasonable division of the study period according to the reservoir water level scheduling. Considering the scheduling mode of the TGR during the rainy season, three different study periods were determined: May (where the reservoir water level drops rapidly), June–August (low reservoir water level) and September (where the reservoir water level rises rapidly). Li *et al*.^4^ have shown that due to the decreased rainfall during the high water level period, and the water pressure maintaining the internal slope, most landslides were stable during this period. In addition, recordings show that only 11.5% of the landslides occurred from October to April of the following year^44^. Therefore, October–April (high reservoir water level) is not included in the study period of this paper.

### Rainfall criteria

To establish an accurate statistical model for rainfall warning, it is necessary to choose the appropriate criteria for defining thresholds^20,31^. A rainfall process can generally be identified by three parameters (rainfall amount, duration and intensity), and we only need two of them to determine most specific rainfall events^44^. Due to daily rainfall data in the study area, we failed to obtain an accurate rainfall intensity. Thus, we elected to use cumulative rainfall amount and duration to identify rainfall events.

To determine the rainfall events most related to landslides, the SPSS 18.0 software (http://www.ibm.com/analytics/spss-statistics-software) was used to calculate correlation coefficients (*R*) between different rainfall events and landslide recordings. As seen in Table 1, the cumulative rainfall of a continuous *n*-day is the most relevant to the occurrence of landslides, but the values of *n* are different in different periods.

**Table 1:** The correlation coefficients between rainfall events and landslide recordings.

Rainfall events (*n* is the duration days of rainfall event before sliding)	correlation coefficient (*R*)		
	May	Jun–Aug	Sep
*n* = 1	0.224	0.362	0.251
*n* = 2	0.200	0.346	0.297
*n* = 3	0.182	0.315	0.208
*n* = 4	0.261	0.282	0.298
*n* = 5	0.187	0.389	0.199
*n* = 6	0.170	0.276	0.204
*n* = 7	0.178	0.289	0.205
Cumulative rainfall in the current month	0.180	0.266	0.216
Cumulative rainfall in the longest rainfall process	0.185	0.242	0.204
Cumulative rainfall in the largest rainfall process	0.216	0.270	0.222

Cumulative rainfall amounts have been widely used^26,30,31^ for defining rainfall thresholds. However, Chang and Chiang^45^ have shown that not all rainfall processes can affect landslide displacement, but only a certain amount of rainfall is able to induce landslide movement. To determine this critical rainfall amount, all earth slides in the study area were selected, and their generalized geological models were established by Geostudio 2012 software (http://www.geoslope.com). The Sigma module in this software was used to complete the landslide stress and strain analysis under rainfall conditions with a specific rainfall amount. The accuracy level of the commonly used GPS equipment (±1–2 mm) was used as the landslide displacement value able to be monitored, and the results showed that most landslides begin to move under rainfall conditions of 30–50 mm, so 30 mm was chosen as the critical rainfall amount inducing landslide movement.

In summary, the determined rainfall criteria in different periods, considering the reservoir water level scheduling, the correlation between rainfall events and landslide recordings, and the response of landslide displacement to rainfall in the study area, are shown in Table 2.

**Table 2:** Rainfall criteria in different periods of landslides in Yunyang County.

Period	Reservoir water level scheduling	Rainfall criteria	
		Rainfall duration	Rainfall amount
May	Decline	Continuous 4 days	Cumulative rainfall exceeding 30 mm
Jun–Aug	Low water level	Continuous 5 days	
Sep	Rise	Continuous 4 days	

### Landslide deformation index

Displacement, displacement velocity, displacement acceleration, and tangential angle are all common landslide deformation indices^46,47^ for black box models. However, according to the comprehensive analysis of severe deformation phenomena and the sudden rapid sliding of typical landslides in the TGRA, the single index of cumulative displacement or displacement velocity cannot be applied to the general situation^48^, and the effect of scale characteristics on displacement needs to be considered.

For landslides with creep deformation, creep displacement is the main deformation component, which is closely related to geotechnical properties (e.g., c, φ, γ,), and the length and thickness of landslides^49^. Therefore, the length of landslides is introduced to represent the landslide scale characteristic (if the landslide is horizontally long, the landslide width is selected), and displacement ratio, which equals the ratio of cumulative displacement to the length of landslide, was used as the statistical index for this study. Compared with other indices, the displacement ratio index is easy to obtain and has high precision because it comes from the monitoring data and basic information of landslides, which meets the requirements of the rainfall warning.

### Statistical analysis

**Figure 5:**
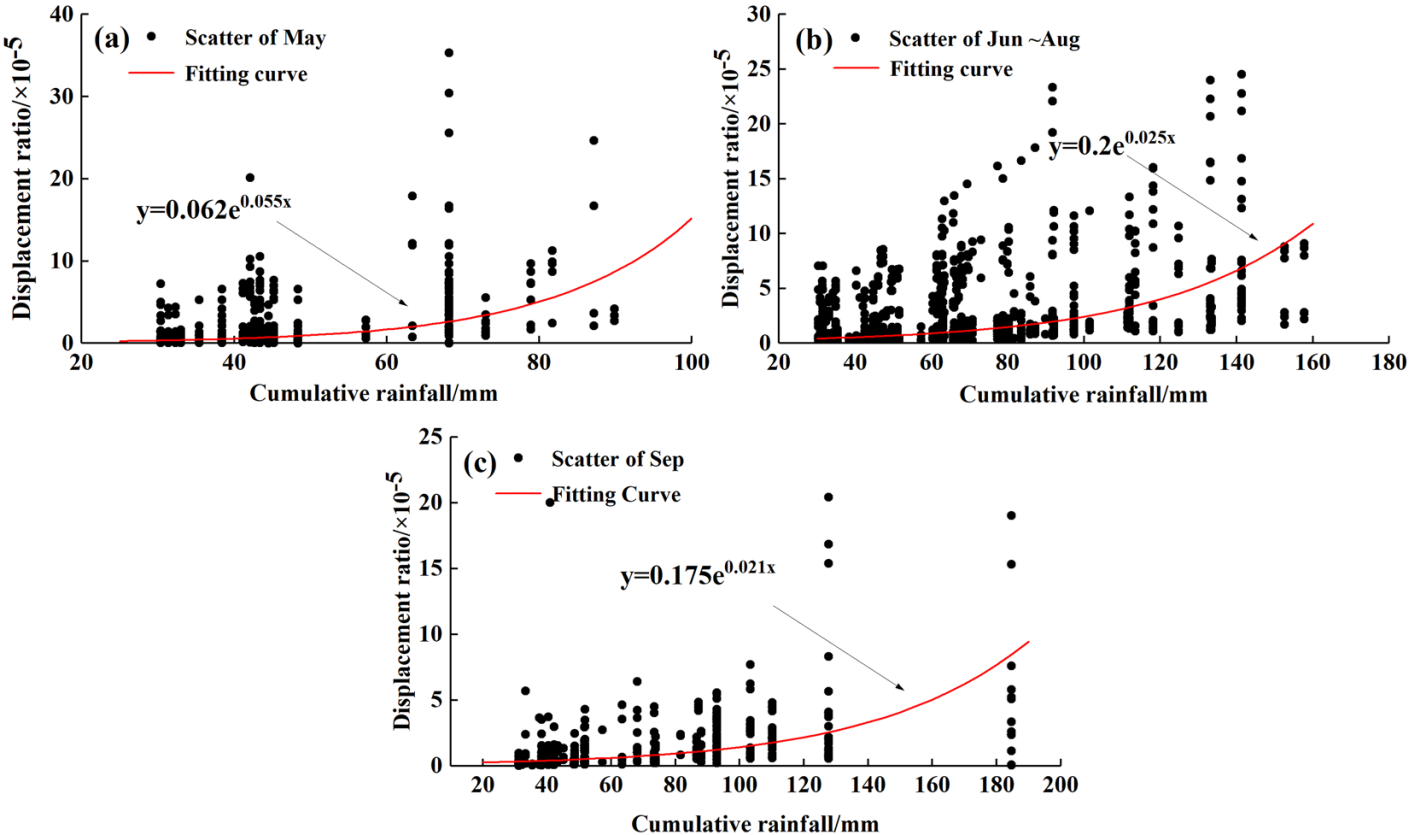
The empirical relationship between displacement ratio and rainfall of landslides in different periods: (**a**) May; (**b**) Jun–Aug; (**c**) Sep.

Due to the limitations of professional monitoring systems, the rainfall data and displacement data usually have differences in time scale. The daily rainfall data and monthly displacement data cannot be used to establish a corresponding relationship, which can make data processing quite difficult. Thus, as seen in rainfall criteria section, a 30 mm rainfall was determined as the critical threshold inducing landslide movement. For a specific rainfall event of the amount of which exceeds this threshold, there should be a positive correlation between the displacement induced by this rainfall processes and the total rainfall amount^18,50^. Certainly, this correlation should be discussed in the same reservoir water level scheduling. In this way, displacement in each month can be decomposed into the displacement of different rainfall events, and the corresponding relationship between monthly displacement data and daily rainfall data can be established. The rainfall criteria values and displacement ratio values of the selected landslides were counted, and the scatter plot between deformation index and continuous *n*-day total rainfall in each period was obtained (Fig. 5). We can see that in a short period of 4–5 days, the growth of displacement ratio is very obvious. So we think there is a statistical relationship between displacement index and rainfall. In most cases, the power function or exponential function can be used to fit their relationship^50,51^. Considering the goodness of fit and previous studies, the letter was determined to obtain the empirical relationship between the displacement ratio and rainfall in different periods, which is as follows:

1 $$\{\begin{array}{lll}\mathrm{May}: & {\rm{y}}={\rm{0.062}}\times {10}^{-5}{{\rm{e}}}^{0.055x} & ({R}^{2}=0.26)\\ {\rm{Jun}}\mbox{--}\mathrm{Aug}: & {\rm{y}}={\rm{0.02}}\times {10}^{-5}{{\rm{e}}}^{0.025x} & ({R}^{2}=0.26)\\ \mathrm{Sep}: & {\rm{y}}={\rm{0.175}}\times {10}^{-5}{{\rm{e}}}^{0.021x} & ({R}^{3}=0.33)\end{array}$$

where *x* is the cumulative rainfall; *y* is the landslide displacement ratio in the rainfall process and *R*^2^ is the goodness of fit. The *R*^2^s in different periods reach nearly 0.3, showing a weak-medium correlation between the fitting curve and the scatter points. For a landslide which is a complex system affected by many factors, single rainfall factor can explain the variability of the landslide displacement ratio of nearly 30%, indicating rainfall is an important factor inducing landslides, but not the only factor. This is consistent with the actual situations in the TGRA. Meanwhile, it is common to directly use statistical black box model to obtain the empirical formula between rainfall and landslide, ignoring internal complex mechanisms^22,50^. Hence, the accuracy of Equation (1) is considered to meet the research need.

### Rainfall thresholds

**Figure 6:**
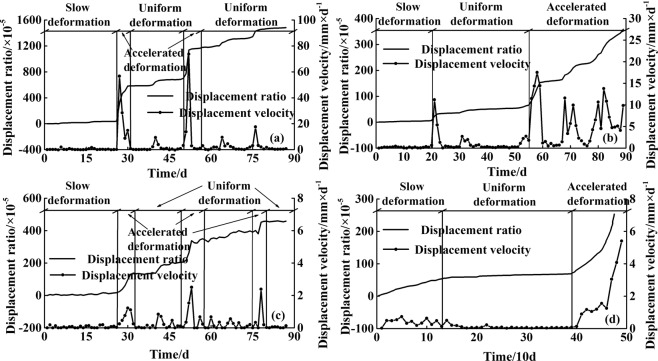
The laws of displacement ratios for some landslides^46,52^: (**a**) Huanglianshu landslide; (**b**) Xintan landslide; (**c**) Zengjiapeng landslide; (**d**) Jimingsi landslide.

The functional relationship between the rainfall criteria and deformation indices has been obtained, and the corresponding rainfall thresholds can be determined by the characteristic values of the displacement ratios. Since all the landslides with implemented professional monitoring systems in Yunyang County are with creep deformation, the monitoring data of all similar landslides that have recently occurred are collected^46,52^, and the displacements in the slow deformation and uniform deformation stages are counted. As seen in Fig. 6 and Table 3, according to the statistics, when the landslide displacement ratio for a certain period reaches 10 × 10^−5^, it can be assumed that the landslide movement has temporarily entered a period of slow deformation or uniform deformation, and a landslide warning is necessary. This value can be considered as the threshold of the deformation index and it was used in Equation (1) (i.e., this value was set as the value of *y*). The corresponding values of *x* are 92.4 mm, 156.5 mm, and 192.6 mm, respectively and they are determined as the rainfall thresholds.

**Table 3:** The displacement ratio values of typical landslides in different deformation stages^18^.

Landslide	Deformation	Displacement in slow deformation stage /mm	Displacement in uniform deformation stage /mm	Length/m	Displacement ratio in slow deformation stage	Displacement ratio in uniform deformation stage
Xintan landslide	Creep	<198	306–3060	2000	<9.9 × 10^−5^	15.3–153 × 10^−5^
Jimingsi landslide	Creep	<39.6	114–380	250	<15.8 × 10^−5^	45.6–152 × 10^−5^
Huangci landslide	Creep	—	50–175	370	—	13.5–47.3 × 10^−5^
Zengjiapeng landslide	Creep	16–29	118–132	610	2.6–4.8 × 10^-5^	19.3–21.6 × 10^-5^
Huanglianshu landslide	Creep	164–224	315–3365	600	27.3–37.3 × 10^-5^	52.5–560.8 × 10^-5^

Among these thresholds, the value in May is the smallest and the value in September is the largest, mainly because reservoir water levels decline rapidly in May, causing a dynamic water pressure difference between the inside and outside of the slope, which is detrimental to slope stability. Under this condition, only a small amount of rainfall combined with the influence of the reservoir water level can induce landslide movement. Conversely, the rising reservoir water level conditions in September are positive for slope stability, so some rainfall is needed to offset this positive impact, causing the rainfall amount that triggers landslide movement to be larger in September than other periods.

## Applying Thresholds to Warning Systems

### CBDRR system in the TGRA

**Figure 7:**
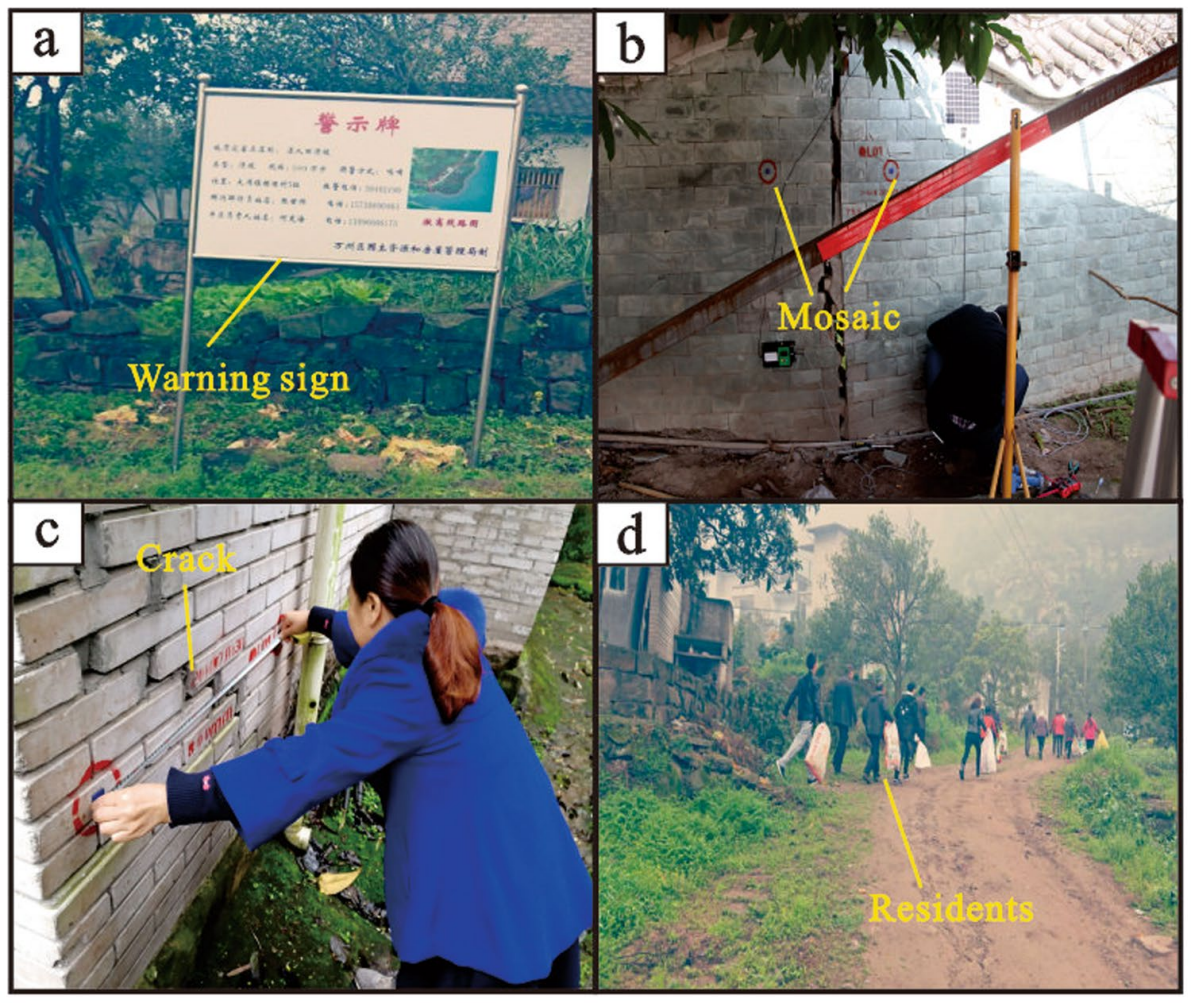
The CBDRR system measures taken in landslides: (**a**) The warning sign marking the basic information of the landslide. (**b**) The building crack monitoring using mosaic. (**c**) The crack monitoring performed by resident. (**d**) The emergency drilling.

To reduce the casualties caused by landslides in the TGRA, a series of structural and nonstructural measures have been taken for significantly threatening landslides. However, a majority of landslides are not covered by those costly measures due to insufficient funds^5^. Thus, the CBDRR system is widely used in the TGRA to reduce landslide risks, especially for the landslides with creep deformation which many people live in (Fig. 7).

The CBDRR is mainly performed by local residents and governments, aiming to find the sliding symptoms and timely evacuate residents through simple monitoring and macro-surveys. Implementation of the system needs in-depth multi-stakeholder cooperation among communities, governments, and experts. Field investigations carried out by experts build a foundation for this customized education and landslide monitoring. A long-term, repeated, customized education for local residents have strengthened their awareness and self-rescue abilities for landslides. Some simple but effective methods are implemented in community-based (CB) landslide monitoring, such as steel piles, mosaic and convergence meters. The CBDRR system has been carried out in the TGRA for more than ten years, and the challenge of the huge number and dispersal of landslides have been gradually overcome^5,15^. Through limited input, the community resilience has improved enormously.

### Warning systems based on rainfall thresholds

Warning systems must be composed of several basic components related to real-time rainfall monitoring and rainfall thresholds^27^. In addition, it is necessary to consider both the risk management and the corresponding measures that could occur during emergency procedures. For example, landslide movement can be identified by both professional monitoring and CBDRR simple monitoring. However, both the data precision and input costs are totally different. If a landslide occurs with slow deformation, the CBDRR method is enough to monitor its deformation trend. Professional monitoring certainly can be performed, but it is not recommended due to the high costs and limited funds. If the landslide deformation clearly accelerates, the professional monitoring is important to help provide a greater understanding of the landslide movement characteristics. Once a landslide is in the most dangerous state, experts and governments must cooperate to discuss emergency measures such as resident evacuation and drilling to reduce risk, and the monitoring system is only an auxiliary method.

**Figure 8:**
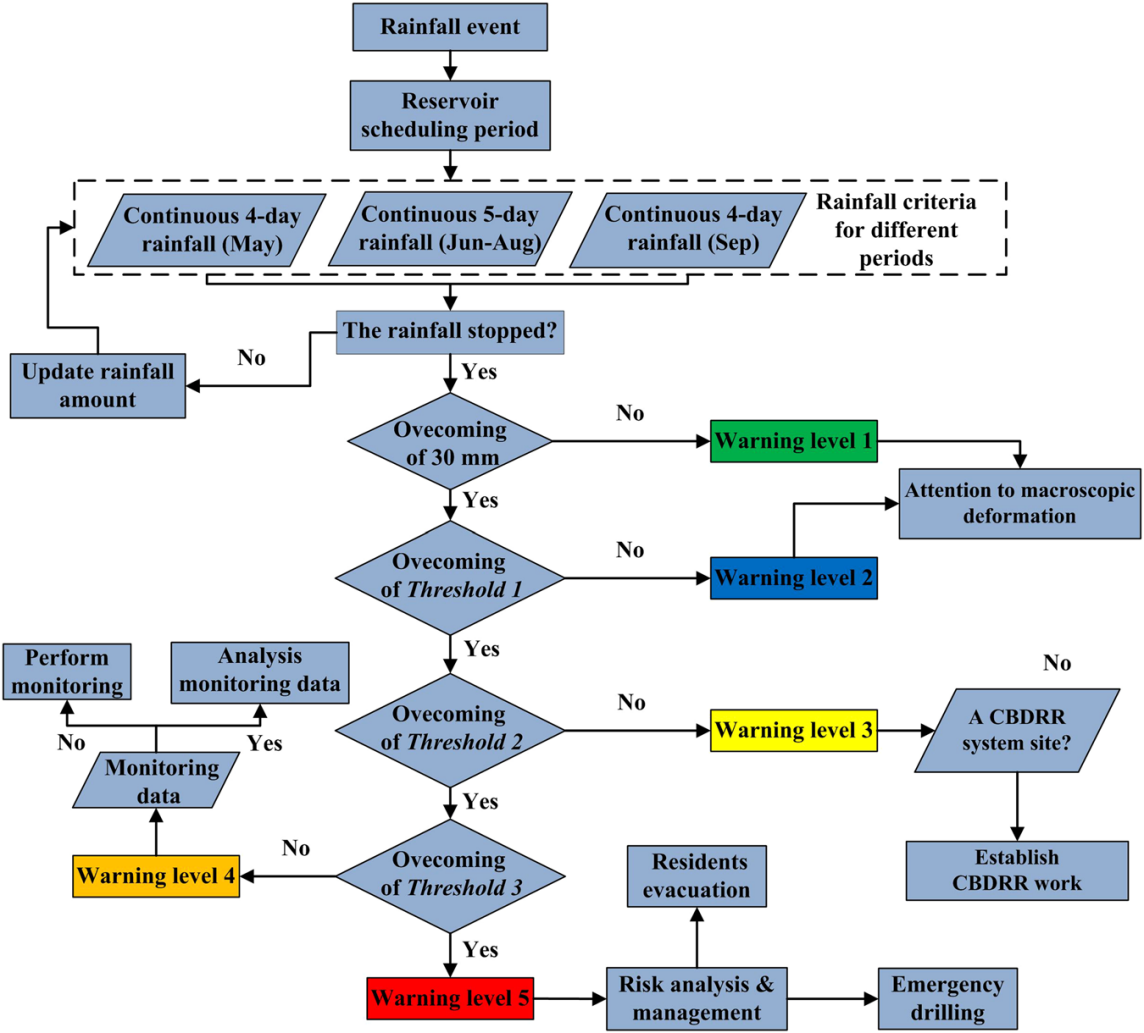
Operating procedure of the warning system based on rainfall thresholds.

From long-term experiences of prevention and control of landslide hazards in the TGRA, we can determine the emergency measures corresponding different warning levels^28^ (Table 4). Among these measures, CBDRR system, professional monitoring and risk management are very common in the TGRA and have been implemented in the field for many years^28,35,37^. Based on this, Fig. 8 describes the operating procedure of the warning system. The procedure is activated when the rainfall events occur. Three thresholds are used to identify the warning level of landslide deformation. In the situation of “warning level 1–2”, it is assumed that the *threshold 1* will not be exceeded and that the landslide occurrence probability is low, so the procedure can be repeated without measures being taken. If the rainfall amount exceeds *threshold 1*, *threshold 2* and *threshold 3* are used to determine the landslide deformation stage and which measures need to be taken. At this stage, the real-time rainfall data has to be obtained and analyzed to estimate the rainfall criterion, i.e., once the rainfall reaches a continuous stage, the real-time rainfall should be considered to update the criterion value by removing earlier data. Thus, if real-time rainfall amounts change sharply, the warning level will dynamically fluctuate, but if rainfall has stopped, the procedure should be stopped. For a long-term rainfall event, we can obtain everyday warning level but it should be noted that the warning level used finally is not the level on the last day but the most dangerous level. So we should calculate all rainfall criteria in this event and elect the largest value to determine final warning level.

**Table 4:** The warning levels of landslides with creep deformation in Yunyang County.

Warning level	Deformation stage	Emergency measures	Rainfall Threshold
1–2	No deformation or very slow deformation	No measures or attention to macroscopic deformation on landslide	92.4 mm (*threshold 1*)
3	Slow deformation	Set up CBDRR system and attention to CBDRR simple monitoring data	156.5 mm (*threshold 2*)
4	Uniform or temporary accelerated deformation	Perform professional monitoring, analyze the monitoring data and make emergency plan	192.6 mm (*threshold 3*)
5	Accelerated deformation and failure	Perform monitoring at full-time; risk management made by government and experts; residents evacuation or emergency drilling	>192.6 mm

## Validation

### Real-time rainfall data warning

In the 2016 rainy season, there were three landslides with a volume greater than 10^5^ m^3^ occurring in the study area:

- A landslide hazard occurred in Longdong Town on June 3^rd^. After the rainstorm, the slope moved slowly for a short time, and the volume reached 1.85 × 10^6^ m^3^. Since this site had been included in the scope of the CBDRR system, the government reminded local residents to pay more attention to the landslide dynamic changes. Finally, the landslide movement stopped, and there was no casualties except for a small number of houses damaged.
- On July 1^st^, a slope in Longjiao Town had a dangerous situation with a volume of 1.9 × 10^5^ m^3^. The disaster management department first evacuated five residents on the slope, and established this slope as a new CBDRR site to closely monitor landslide development after the accident was handled.
- A landslide clearly developed in Fengming Town on July 1^st^, with a volume of 3 × 10^5^ m^3^. The slope was not inhabited, and the relevant departments began CBDRR work at this site.

**Figure 9:**
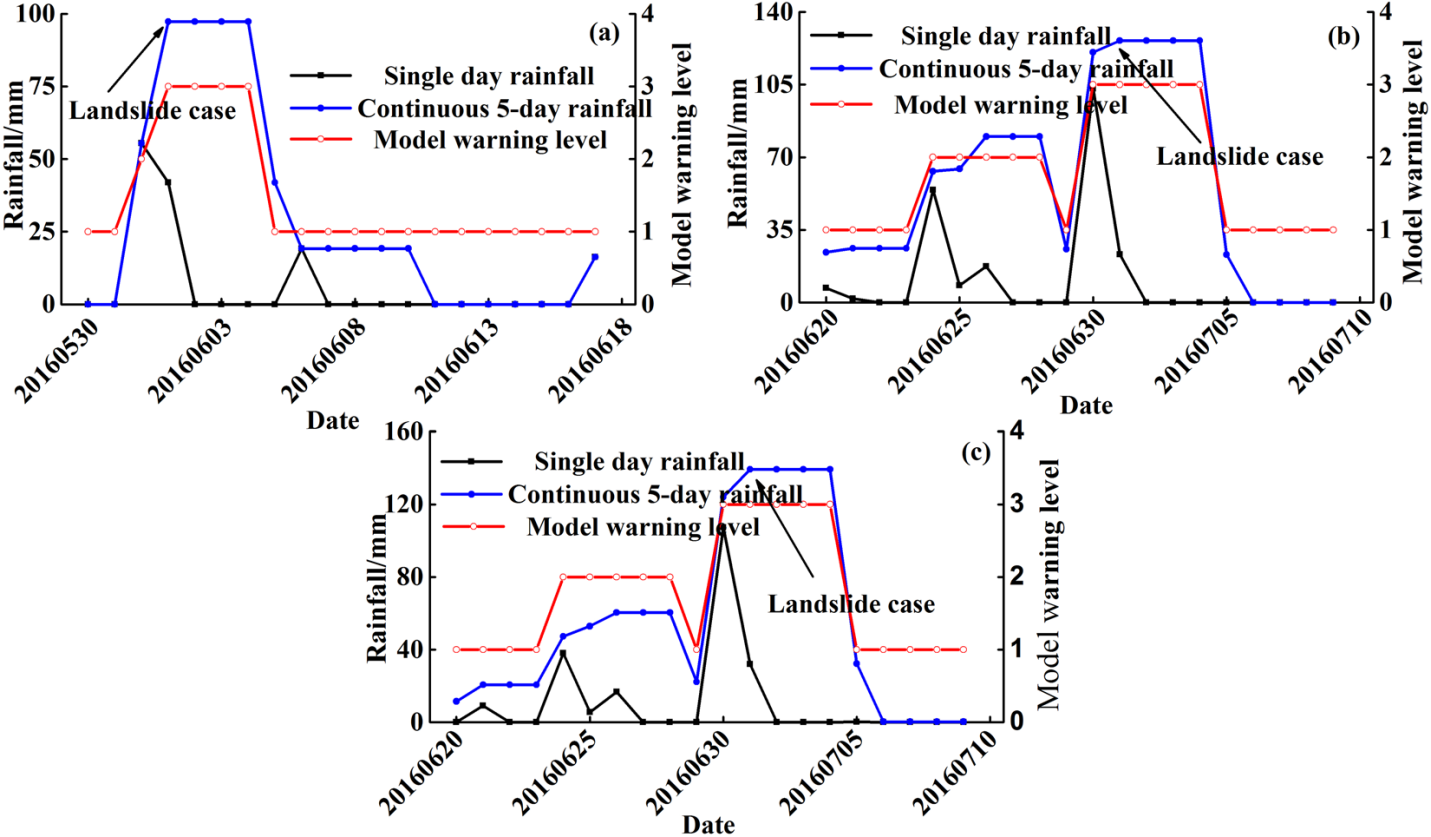
The comparison between model warning results and actual situations of three landslide cases in 2016: (**a**) Landslide case in Longdong Town; (**b**) Landslide case in Longjiao Town; (**c**) Landslide case in Fengming Town.

As seen in Table 5, the rainfall criteria values of the above cases were obtained from rainfall data statistics, and the predictive warning levels are highly consistent with the actual response measures. In addition, rainfall criteria values within 20 days of the occurrence of these cases and their corresponding warning results were compared (Fig. 9). The results indicated that the warning levels on the dates when the landslides occurred were the most dangerous levels during the 20-day periods. Meanwhile, compared to the level on the dates when the dangerous situations occurred, warning levels within 1–3 days of the case were safer, which proves that the model is feasible and can produce highly accurate predictive results.

**Table 5:** Statistical information of Yunyang County landslides in 2016.

Date	Location	Rainfall criterion/mm	Predictive warning level	Actual disposal measures
2016.6.3	Longdong Town	97.3	Warning level 3	More attention to simple monitoring data (The CBDRR has been performed)
2016.7.1	Longjiao Town	126.2	Warning level 3	Performed emergency drilling and began the CBDRR system after the event
2016.7.1	Fengming Town	139.3	Warning level 3	Began the CBDRR system in the site

### Deformation monitoring data warning

**Figure 10:**
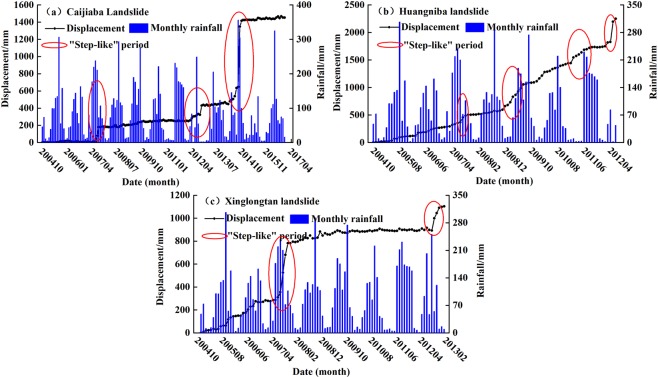
The step-like displacement curves of typical landslides with creep deformation in Yunyang County: (**a**) CJBL; (**b**) HNBL; (**c**) Xinglongtan landslide (XLTL).

The landslide monitoring data in Yunyang County are detailed and adequate, which provides a strong basis for providing rainfall warnings. Landslide displacement-time curves can visually show the landslide movement characteristics under rainfall conditions. Three landslides with professional monitoring system were selected in the study area, all of which have similar “step-like” displacement-time curves (Fig. 10). As seen in Table 6, the landslide deformation index and rainfall criterion corresponding to each “step” period are counted, and the results show that during each landslide displacement “step” period, the predictive warning results are at least warning level 3 (or very close), and some even reach warning level 4. All the corresponding displacement ratio values exceed the characteristic value of 10 × 10^−5^ (except for the “step” period of CJBL in 2012), and the ratio of these values to the total landslide displacement during this period is mostly up to 5% or even 10%. The monitoring reports provided by the relevant institutes show that the sudden increase of landslide displacement in the actual situations has caused economic losses such as damage to houses. Corresponding measures such as relevant resident evacuation and the strengthening of monitoring data collection have been adopted for landslide risk reduction. Overall, the warning results of landslide professional monitoring data are consistent with the actual conditions, indicating the model prediction accuracy is good.

**Table 6:** The warning results of some landslide monitoring sites with “step-like” behavior.

Landslide	Step-like period	The largest rainfall criterion value /mm	Predictive Warning level	Actual displacement /mm	Displacement ratio/×10^–5^	Percentage of total displacement /%
CJBL	2007.6~2007.8	133.5	Warning level 3	167.1	11.1	11.5
	2012.6~2012.9	91.8	Warning level 2	123.7	8.2	8.5
	2014.8~2014.9	133.1	Warning level 3	701.8	46.8	48.3
HNBL	2007.6~2007.8	133.5	Warning level 3	103.9	23.1	4.6
	2009.5~2009.9	184.6	Warning level 4	157.3	35.0	7.0
	2011.5~2011.8	157.7	Warning level 4	82.4	18.3	3.7
	2012.5~2012.8	104.8	Warning level 3	147.9	32.9	6.6
XLTL	2007.6~2007.8	133.5	Warning level 3	325.5	27.1	29.5
	2012.6~2012.9	91.8	Warning level 2	147.1	12.3	13.3

## Discussion

The displacement ratio was taken as the deformation index in this paper while previous studies generally chose the displacement or velocity. Although previous applications are few, the scale characteristics of landslides can be considered, and the scale effects of different landslide displacements can be offset to the greatest extent. As seen in Equation (2), the scatter fitting formula is obtained by the landslide displacement index. The displacement threshold is determined to be 30 mm and the final rainfall thresholds are 107 mm, 163.4 mm and 258.2 mm, respectively. If the displacement velocity index is used, the class of “slow” can be the characteristic value of 4.38 mm/d^41,42^. The rainfall thresholds are 103.4 mm, 190 mm and 221.7 mm, respectively. Hence, the rainfall thresholds calculated by displacement and displacement velocity indices are significantly larger than the results of this study, indicating the warning results obtained by the displacement ratio model are more conservative than the results obtained using the displacement model and displacement velocity model.

2 $$\{\begin{array}{ll}\mathrm{May}: & y=2.303{{\rm{e}}}^{0.024x}\\ {\rm{Jun}}\mbox{--}\mathrm{August}: & y=3.048{{\rm{e}}}^{0.014x}\\ \mathrm{September}: & y=4.923{{\rm{e}}}^{0.007x}\end{array}$$

3 $$\{\begin{array}{ll}\mathrm{May}: & y=0.034{{\rm{e}}}^{0.047x}\\ {\rm{Jun}}\mbox{--}\mathrm{August}: & y=0.081{{\rm{e}}}^{0.021x}\\ \mathrm{September}: & y=0.101{{\rm{e}}}^{0.017x}\end{array}$$

The low R^2^ values in Equations (1)~(3) represent a poor correlation, which indicate that a black box model using only rainfall is not very effective in our work. This means that: (i) our results should be considered only preliminary and need further studies before setting up a reliable warning system; (ii) rainfall cannot be considered the only explanatory variable: it is clear that other factors (water level, creeping processes, geomorphological setting…) play a very important role in conditioning the displacement rate.

In summary, adequate monitoring data is the key to rainfall warning system. The model established in this paper applies a large number of landslide professional monitoring data which were obtained from on-site landslide monitoring system by ourselves. Due to detailed data, the model has a reliable reference value and it can be used not only to obtain the rainfall thresholds but also to obtain the five warning levels for landslides with creep deformation. Meanwhile, the model considers landslide scale characteristic in the study area and historical landslide events caused by rainfall, making final warning results closer to the actual situations. However, because most velocities data used in the paper are very slow or extremely slow, more tests are needed to validate the warning reliability in the presliding stage (warning level 5) of landslides. Meanwhile, subjected to the monitoring system of TGRA, temporal resolutions of displacement data is not daily but monthly. Although the monthly displacement data can be considered detailed enough for very slow and extremely slow landslides, more displacement data with daily temporal resolution is needed for more accurate rainfall thresholds.

## Conclusion

There are many landslides with creep deformation in the TGRA. They generally experience sudden accelerations triggered by heavy rainfall. Taking Yunyang County in the TGRA as an example, a black box model was proposed to perform rainfall warning of landslides with creep deformation at a regional scale. In this process, based on developed landslide monitoring systems, the model takes the landslide displacement ratio as the deformation index, which considers the landslide scale characteristic, and establishes the empirical correlation between monthly landslide surface displacement monitoring data and daily rainfall data. The cumulative rainfall thresholds that were determined relied on this model and were then applied within a warning system to obtain the five-level warning partition for the study area. The landslide cases and professional monitoring data were used to validate the model accuracy. The results indicate that the predictive warning results are consistent with the actual situations.

Overall, adequate monitoring data and reasonable deformation index are the keys to rainfall warning system. This study provides an experience for the analysis of monitoring data and a reliable choice for the deformation index. Meanwhile, different from previous research efforts about presliding warnings, the method proposed in this paper, which applies statistic theory and black box model, can achieve accurate warning for landslides with creep deformation and it can be recommended to conduct regional landslide early warning in the TGRA and other landslide-prone regions.

## Data Availability

The relevant datasets in this study are available from the corresponding author on reasonable request.
